# Synthesis of a novel ternary ZIF-8/GO/MgFe_2_O_4_ nanocomposite and its application in drug delivery

**DOI:** 10.1038/s41598-021-98133-2

**Published:** 2021-09-21

**Authors:** Saleheh Sanaei-Rad, Mohammad Ali Ghasemzadeh, Seyyed Mohammad Hossein Razavian

**Affiliations:** 1 Department of Chemistry, Qom Branch, Islamic Azad University, Qom, Islamic Republic of Iran; 2 Department of Microbiology, Qom Branch, Islamic Azad University, Qom, Islamic Republic of Iran

**Keywords:** Biochemistry, Microbiology, Medical research, Chemistry, Nanoscience and technology

## Abstract

In recent year, metal–organic frameworks (MOFs) have been displayed to be a category of promising drug delivery systems because of their crystalline structure, the potential of further functionality, and high porosity. In this research, graphene oxide was synthesized from pure graphite via hummer method and then MgFe_2_O_4_ nanoparticles was incorporated into the synthesized ZIF-8 metal–organic frameworks which followed with loading on the surfaces of graphene oxide. In continue, tetracycline as an antibiotic drug was loaded on the surfaces and the cavities of the prepared nanocomposite. The outcomes of this research revealed that 90% of the tetracycline was loaded on the synthesized ZIF-8/GO/MgFe_2_O_4_ nanostructure. Next, drug release was done at pH: 5 and pH: 7.4 within 3 days, resulting about 88% and 92% release of the tetracycline, respectively. With using different spectroscopic methods like X-ray crystallography (XRD), scanning electron microscope (SEM), energy-dispersive X-ray spectroscopy (EDX/Mapping), Fourier transform infrared (FTIR), thermalgravimetric analysis (TGA), and Brunauer–Emmett–Teller (BET), the structure of synthesized materials was confirmed. Furthermore, the antibiotic activity of tetracycline trapped into the ZIF-8/GO/MgFe_2_O_4_ was evaluated by agar-well diffusion method on both gram-positive (*Staphylococcus aureus*) and gram-negative (*Escherichia coli*) bacteria, which showed good antibacterial results.

## Introduction

In last decade, more attention has been paid to improvement novel techniques for the advancing of drug delivery systems. The increasing numbers of drug resistances in bacterial contaminants have become important healthcare challenges due to the severe reduction in the number of therapy and repetitive treatment accessible. This issue has led to enhanced morbidity developed medicinal. As a result, combination treatment involving the co-application of existing antibiotics with unique nano structures such as metal halides, nano-sized transporters, and metal oxide nanoparticles is a promising approach to counter antimicrobial opposition^1^.

**Figure 1:**
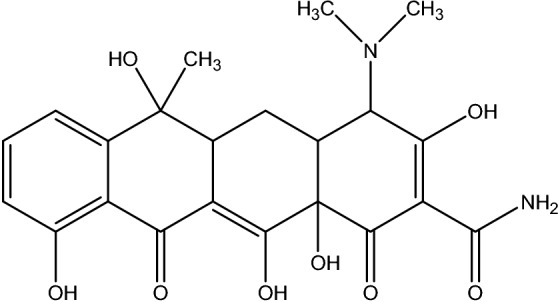
Chemical structure of tetracycline.

The tetracycline (TC) is a type of antibiotics that constrain protein combination by stopping the addition of aminoacyl-tRNA to the ribosomal acceptor (A) sites (Fig. 1)^2^.

TCs are broad-spectrum agents, displaying activity against a varied kind of bacteria^3^. The favorable antimicrobial and antifungal attributes of these agents and the loss of mighty adverse side effects has led to their applications in the treatment of animal and human infections. The main feature of this drug is its use at certain hours in order to completely control the infection^2^.

Nano-scale carbon allotrope have been extensively investigated by researchers for targeted drug delivery. In this regard, graphene sheets with highly active surfaces are astonishingly desirable for fundamental research and technological applications. This two-dimensional structure has been considered due to having several functional groups connected to carbon plates with high density, biocompatibility, high electrical, and conductivity properties^4^.

Graphene oxide (GO) as a kind of graphite derivatives give easy dispensability in water and other organic solvents. GO possess wealthy oxygen containing functionalities such as epoxide, carboxyl, ketone, lactone, and hydroxyl groups^5^. Furthermore, it has a great specific surface area and displays excellent activities which are a versatile tool in chemical transformations^6^, and also in drug delivery systems^7^.

Metal–organic frameworks have lately concerned very attention in the meantime. MOFs display many basic specifications in drug delivery due to flexible building, variable sizes and figures, easily functionalization, high porosity, easy biodegradability and high design capability^8–10^. ZIF-8 is a type of zeolitic imidazole frameworks (ZIFs) which have excellent benefits because of highly pore sizes, massive surface area and simple variation of the pore sizes inside the frameworks with modifiable organic groups^11^. However, to improve the stability of the carrier, scientists have been used some metals with high oxidation state (Ni^2+^, Al^3+^, Zr^4+^, Fe^3+^, Mg^2+^) to form powerful coordination bond in ligands to provide extremely stable MOFs. Therefore, great oxidation state metal-based MOFs or their composites display considerable potential in antibiotic sensing^12,13^.

We used MgFe_2_O_4_ nanoparticles with crystal structure in which the particles are rather disordered than crystalline particles, there by having many more defects can provide better nanocarrier concentration and extra active sites. Also, magnetic nanoparticle MgFe_2_O_4_ exhibited greater super paramagnetic propriety and homogeneous diameter. This information indicates that Mg (II) is safe and practical to use in a drug delivery systems^14^.

Previously, metal–organic framework skeletons have been used as potential nanocarriers in drug delivery of some antibiotics^1,15^, folic acid^16^, anticancer drugs^17,18^ and ibuprofen^19^.

Considering the significance of the previous issues associated to discovery of new drug delivery systems to find a suitable and practical nanocarrier^20–22^, we were capable to introduce a unique and impressive nanostructure like ZIF-8/GO/MgFe_2_O_4_ as a carrier for the loading and release of TC.

**Figure 2:**
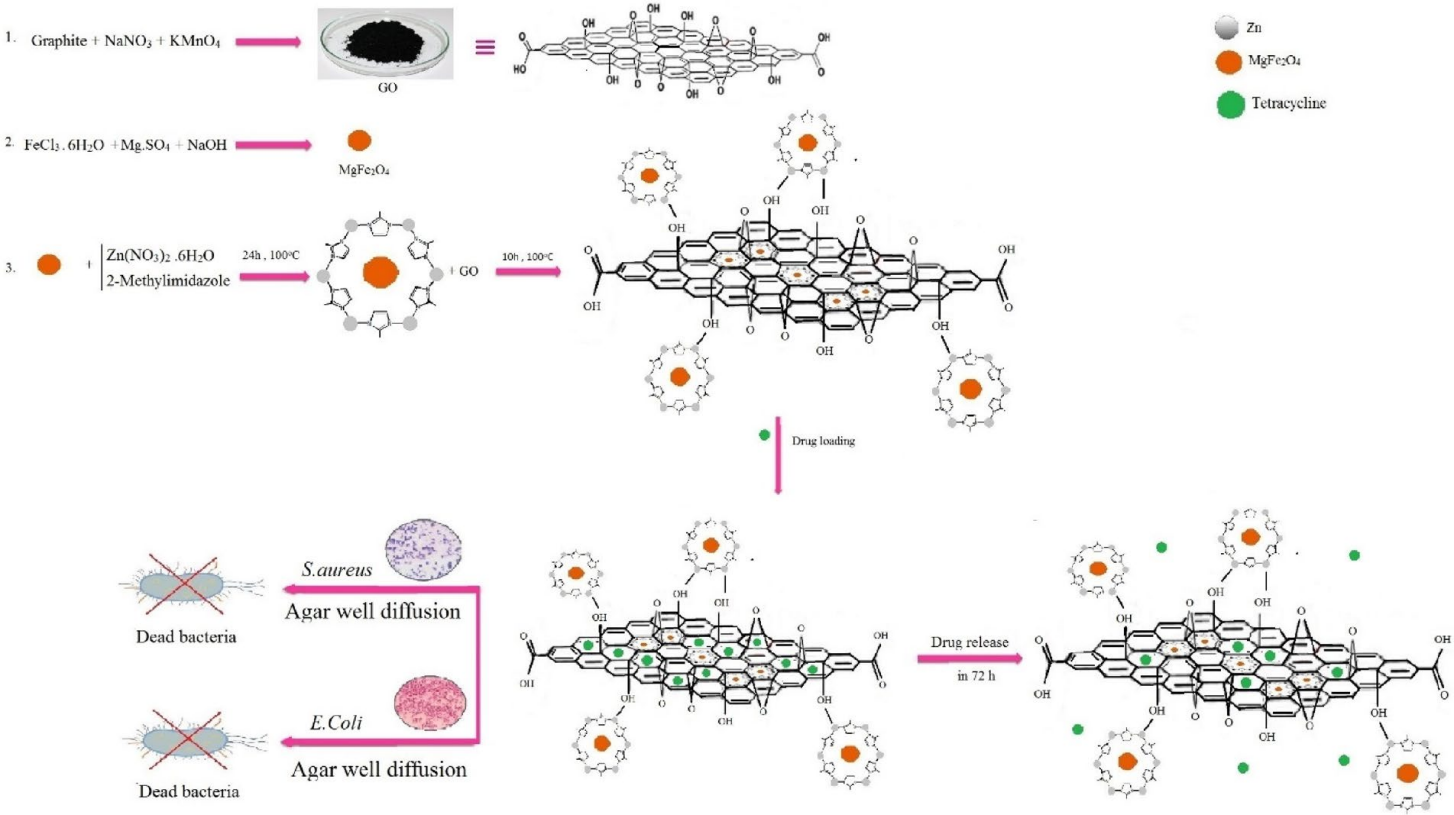
Simulation of the preparation of the ZIF-8/GO/MgFe_2_O_4_ framework, drug loading, release and antibacterial tests.

Furthermore, the antibacterial activities of the prepared composites were compared and evaluated with pure tetracycline against *Staphylococcus aureus* and *Escherichia coli* bacteria by agar-well diffusion method which exhibited considerable results. The target of the this work was to progress the performance of the antibacterial drug delivery method, and drug release control (Fig. 2).

## Experimental

### Materials and instruments

Chemicals materials were bought from the Sigma-Aldrich and Merck in high purity. FT-IR spectra were recorded on Magna-IR spectrometer 550. Powder X-ray diffraction (XRD) was carried out on a Philips diffractometer of Xˈpert Company with mono chromatized Cu kα radiation (λ = 1.5406 Ả). The compositional analysis was done by energy dispersive analysis of X-ray (EDX, Kevex (Newark, DE) Delta Class I). Microscopic morphology of products was visualized by SEM (LEO1455VP). Thermogravimetric analysis (TGA) was performed on a Mettler Toledo TGA under argon and heated from room temperature to 825. The approximate sample weight was 10 mg in TG experiment with 10 °C/min heating rate. Nitrogen adsorption–desorption isotherms were measured at 196 using a Belsorp mini automatic adsorption instrument after degassing the samples at 150 °C for 5 h. Absorption spectra were recorded in the range 200–800 nm on a Shimadzu model.

1601 PC UV–visible spectrophotometer (Shimadzu, Tokyo, Japan).

### Synthesis MgFe_2_O_4_ nanoparticles

A mixture of MgSO_4_ (1.2 g) and FeCl_3_.6H_2_O (2.4 g) were dissolved in deionized water (30 mL) was stirred until to afford a clear mixture (Solution A). Then, 3.2 g of NaOH was solved in 15 mL of deionized water by a magnetic stirrer, this solution was labeled as mixture B. Then, the solution B was gradually added to the solution A, which followed under vigorous stirring. Eventually, the solution was transferred in stainless steel pressure vessel. The autoclave was kept at 180 °C for 10 h and then cooled down slowly. The obtained residue was collected, washed three steps with deionized water, dried and then calcinated in a furnace at 700 °C for 3 h^23^.

### Preparation of ZIF-8/MgFe_2_O_4_

In a backer, MgFe_2_O_4_ NPs (0.5 g) and Zn(NO_3_)_2_.6.H_2_O (0.6 g) were mixed in methanol (10 mL). In another vessel, 2-methyl imidazole (1.3 g) was poured in methanol (10 mL) and then the mixture was stirred vigorously. Then, two vessels were mixed together and the solution was mixed completely for 30 min. The obtained residue was separated by centrifugation method and was washed several times with methanol. Eventually, the product was dried at 100 °C for 24 h^24^.

### Synthesis of graphene oxide

GO was prepared by Hummer's method. Normally, pure graphite (5 g) and sodium nitrate (2.5 g) was added to sulfuric acid (115 mL, 98%) and the solution was equipped to a magnetic stirrer with a condenser which placed in an ice bath. During the stirring of the mixture, KMnO_4_ (15 g) was added slowly for 120 min.

The mixture was placed in a water bath (35 °C) and stirred for half hour. Next, 230 mL of ultrapure water was gradually added into the mixture and stirring was continued at 98 °C for 15 min. In continue, 700 mL of DI water and H_2_O_2_ (50 mL, 30%) were respectively added to the solution to end the reaction. When the reaction completed, the product was washed with HCl (5%) and DI water for three times. The obtained GO was dried at 60 °C for 12 h^25^.

### Preparation of ZIF-8/GO/MgFe_2_O_4_

A mixture of 0.1 g of ZIF-8/MgFe_2_O_4_ nanocomposite and GO (0.5 g) was dispersed in DMF (30 mL) under ultrasonic irradiations and then the mixture was conducted into an autoclave at 100 °C for 10 h. When the reaction was ended, the obtained residue was separated and washed three several times with dimethyl formamide, DI water and ethanol, respectively. Lastly, the prepared ZI F-8/GO/MgFe_2_O_4_ was dried at 40 °C for overnight.

### Encapsulation of tetracycline into the ZIF-8/GO/MgFe_2_O_4_

In order to drug loading on ZIF-8/GO/MgFe_2_O_4_, 0.05 g of the activated ZIF-8/GO/MgFe_2_O_4_ powder was mixed with tetracycline (0.1 g) and ultrapure water (5 mL). The suspension solution of ZIF-8/GO/MgFe2O4/TC was stirred for the duration of 5 days at room temperature. Then, the product including tetracycline encapsulated on nanocomposite was separated by centrifuge and washed two times with H_2_O and EtOH. Finally, the sediment was dried at 40 °C for 24 h. The amount of encapsulated drug was 90% based on the Eq. (1).

1 $$ {\text{Drug loading }}\left( \% \right)\, = \,\left( {{\text{TC weight in example}}/{\text{total weight of example}}} \right)\, \times \,{1}00\% $$

### Drug release from ZIF-8/GO/MgFe_2_O_4_/TC

The release of tetracycline was investigated in buffer acetate and phosphate buffer saline. In this method, ZIF-8/GO/MgFe_2_O_4_/TC (0.02 g) was added to the 50 mL of every buffer, individually. Next, the mixture was stirred at 37 °C for 72 h. Every time, 5 mL of the mixture was removed and replaced with the equal value of the fresh buffer. The value of tetracycline release from the ZIF-8/GO/MgFe_2_O_4_/TC composite was checked by UV–Vis spectrometer at 360 nm. It was demonstrated that the drug release according to Eq. (2) was achieved 92% and 88% at pH: 5, and pH: 7.4, respectively.

2 $$ {\text{Release percentage }}\left( \% \right)\, = \,{\text{mr }}\left( {\text{amount of released TC}} \right)/{\text{ml }}({\text{total amount of loadedTC}}). $$

### Antibacterial studies

The antimicrobial activity of the pure TC, ZIF-8/GO/MgFe_2_O_4_, and ZIF-8/GO/MgFe_2_O_4_/TC were measured via the agar-well diffusion technique, that fresh cultures of *Escherichia coli* (*E. coli*), and *Staphylococcus aureus* (*S. aureus*), bacteria were used. Initially, Moller agar (40 mL) was incubated into every sterilized Petri plate with the analogous strains of bacteria. The plates were left to solidify at room temperature for 1 day. Afterward, a well of 6 mm diameter was done by a sterile cork borer. Next, 50 and 100 μl of tetracycline, ZIF-8/GO/MgFe_2_O_4_, and ZIF-8/GO/MgFe_2_O_4_/TC which was prepared at a concentration of 10 mg/mL of HCl was transferred in diameter well. Finally, the plates were incubated for overnight at 37 °C.

## Results and discussion

### FT-IR spectroscopy

**Figure 3:**
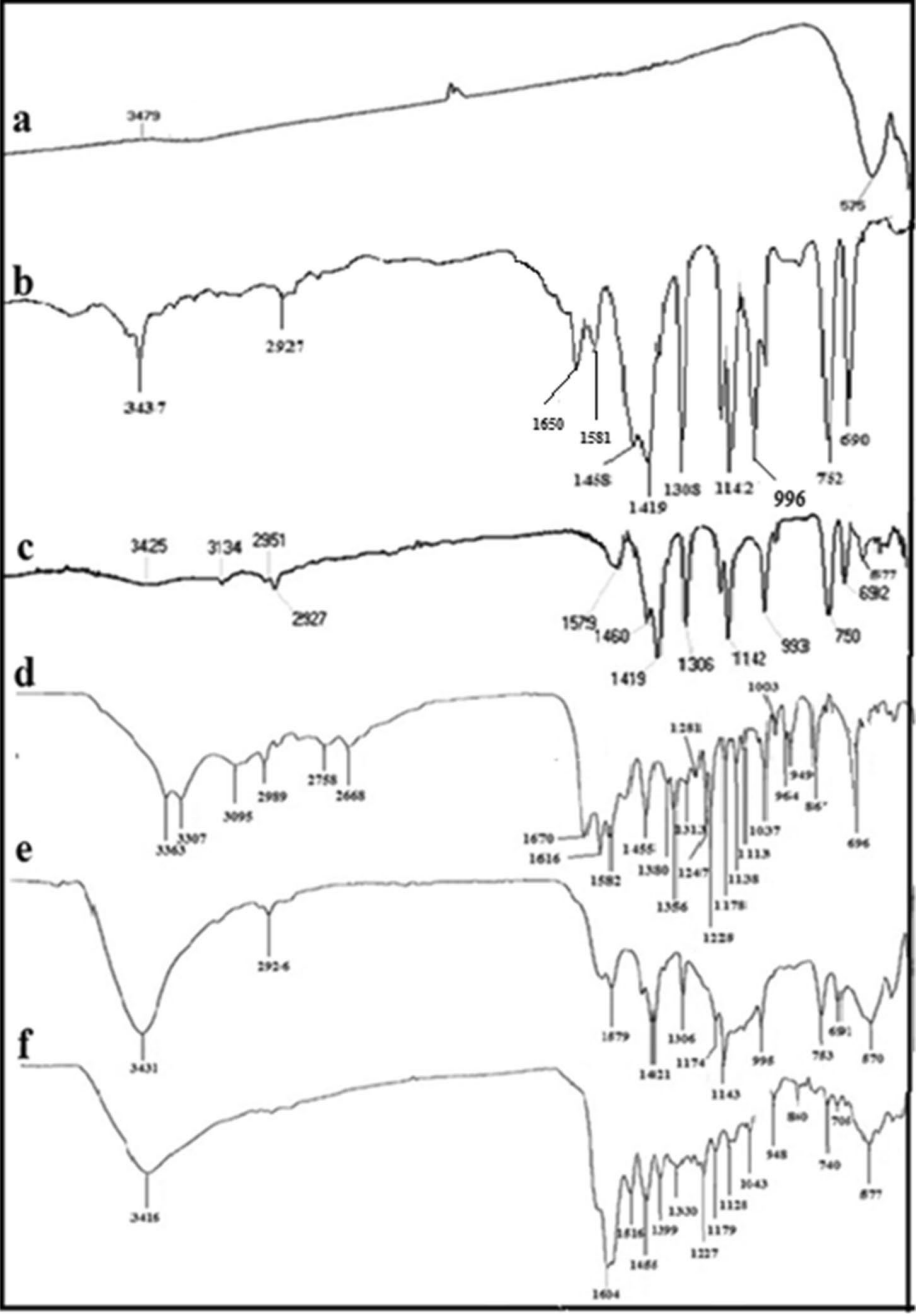
FT-IR spectra of MgFe_2_O_4_ **(a)**, ZIF-8 **(b)**, ZIF-8/MgFe_2_O_4_ **(c)**, TC **(d)**, ZIF-8/GO/MgFe_2_O_4_ **(e)**, ZIF-8/GO/MgFe_2_O_4_/TC **(f)**.

The FT-IR spectra of MgFe_2_O_4_, ZIF-8/MgFe_2_O_4_, tetracycline, ZIF-8/GO/MgFe_2_O_4_, and ZIF- 8/GO/MgFe_2_O_4_/TC are shown in Fig. 3. The absorption bonds related to Mg–O and Fe–O in MgFe_2_O_4_ nanoparticles are appeared at 435 cm^−1^, and 575 cm^−1^, respectively (Fig. 3a)^23^.

FT-IR spectrum of ZIF-8 is shown in Fig. 3b. As indicated the corresponding absorption bands at 485, 1180, 1581, 1650, and 2927 cm^−1^ are corresponding to Zn–N, C–N, C=C, C=N and C−H, respectively. Figure 3c displays FT-IR spectrum of ZIF-8/MgFe_2_O_4_ composite. As illustrated, the stretching vibrations due to Mg–O and Fe–O in MgFe_2_O_4_ NPs are appeared at 435 and 575 cm^−1^. Moreover, the main absorption bonds in ZIF-8 structure are appeared at 2925, 1589 and 485 cm^−1^ due to stretching vibrations of C−H, C=N and Zn–N bonds, respectively^26^.

FT-IR spectrum of the pure tetracycline is indicated in Fig. 3d. As indicated, the corresponding peaks because of the presence of OH and NH_2_ groups were observed at 3363 and 3305 cm^−1^. Also, The peaks showed the C–H stretching vibrations of aromatic rings from 3095 to 2989 cm^−1^, C=C stretching from 1582 to 1616 cm^−1^, and C–N stretching at 1228 cm^−1^, C=O stretching at 1670 cm^−1^, and C–O stretching from 1037 to 1113 cm^−1^ ^27^. The FT-IR spectrum of ZIF-8/GO/MgFe_2_O_4_ displays a broad and intense peak at 3367 cm^−1^ due to stretching vibration of O−H groups in grapheme oxide. The absorption bonds at 1724 cm^−1^ is related to the stretching vibration of carbonyl group at the edges of GO. Whereas, the peaks at 1045 cm^−1^ and 1215 are due to the stretching vibrations of C-O bands (Fig. 3e)^23^.

Figure 3f displays the FT-IR spectrum of ZIF-8/GO/MgFe_2_O_4_/TC which approves the existence of each component in the structure. The stretching vibrations of Mg–O and Fe–O in MgFe_2_O_4_ NPs are shown at 435 and 575 cm^−1^. The peak at 3367 cm^−1^ is related to the OH groups. The absorption band at 3305 cm^−1^ depends to NH_2_ moiety of TC, representing the successfully encapsulation of TC into the structure. The absorption bonds at 1724 cm^−1^, 1045 cm^−1^, and 1215 cm^−1^ is related to the stretching vibration of carbonyl group, and C–O bonds in the structure of GO.

### Field-emission scanning electron microscope analysis

**Figure 4:**
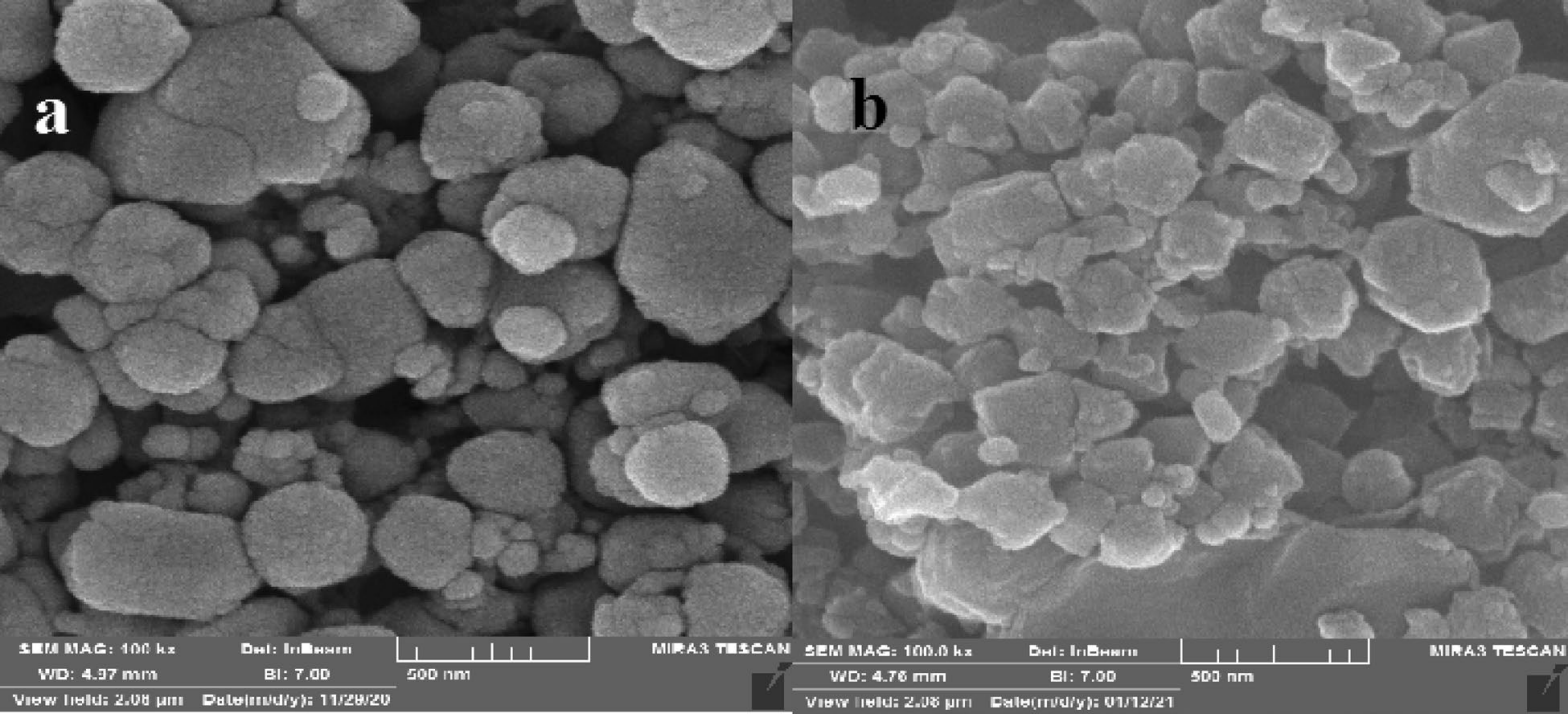
SEM images of ZIF-8/GO/MgFe_2_O_4_ **(a)**, and ZIF-8/GO/MgFe_2_O_4_/TC **(b)**.

The particle size and morphology of the ZIF-8/GO/MgFe_2_O_4_ before and after TC loading are indicated in Fig. 4. SEM illustration of the ZIF-8/GO/MgFe_2_O_4_ shows that nanoparticles have almost spherical structure (Fig. 4a). In Fig. 4b, with loading of the TC on the surfaces of the ZIF-8/GO/MgFe_2_O_4_ nanocomposite, the particle size was increased and also the morphology of the nanocomposite disordered due to drug loading.

### Energy-dispersive X-ray spectroscopy

**Figure 5:**
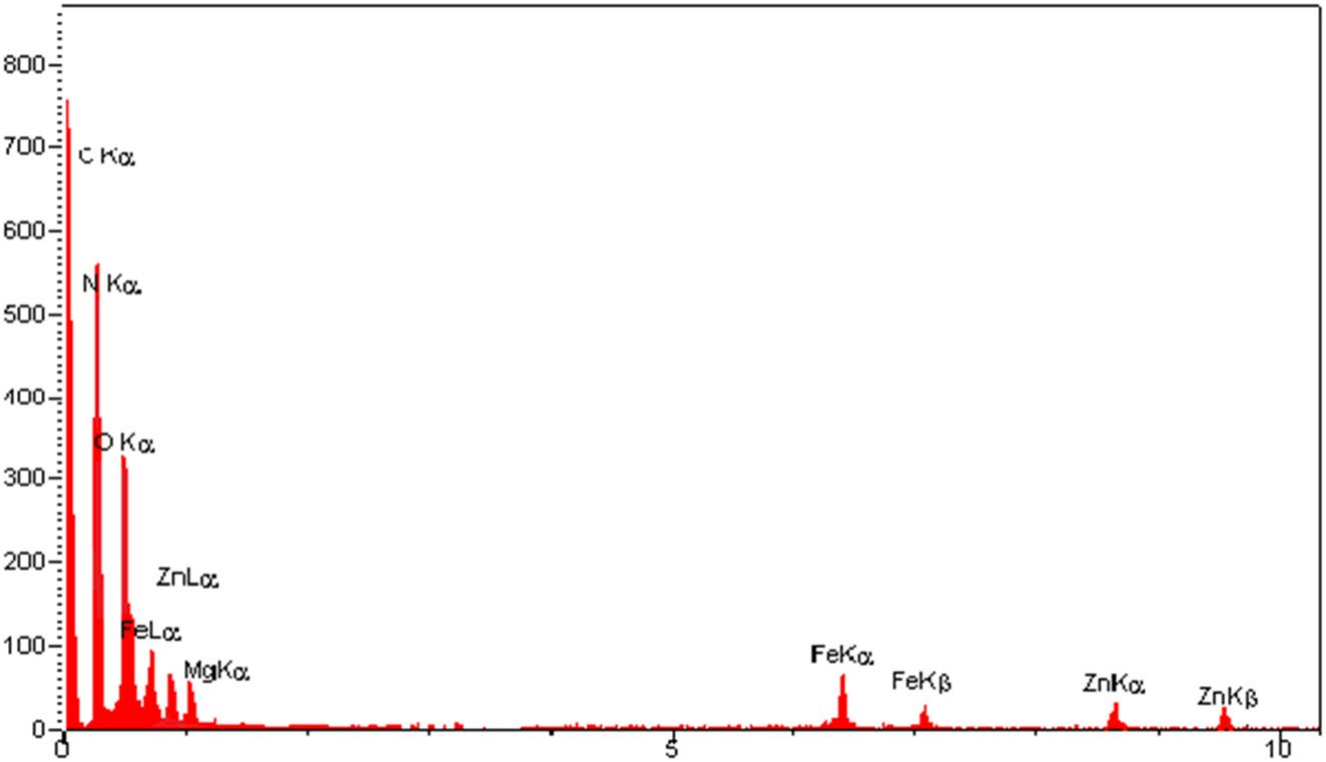
EDX spectrum of ZIF-8/GO/MgFe_2_O_4_/TC.

EDX technique was applied to explore the elemental components of the prepared ZIF-8/GO/MgFe_2_O_4_/TC nanocomposite. Figure 5 shows that the elements are including Mg, O, Fe, C, N, and Zn for the ZIF-8/GO/MgFe_2_O_4_/TC nanostructure.

**Figure 6:**
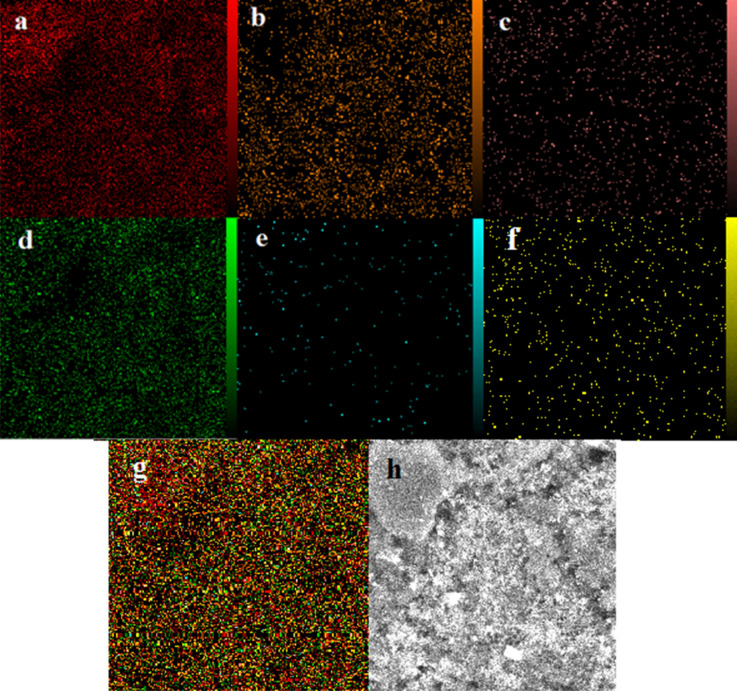
EDX-Mapping of the C **(a)**, Fe **(b)**, N **(c)**, O **(d)**, Zn **(e)**, Mg **(f)**, ZIF-8/GO/MgFe_2_O_4_/TC **(g)**, SEM images in 10 μm **(h)**.

Furthermore, EDX analysis has been performed in the mode of elemental mapping of the ZIF-8/GO/MgFe_2_O_4_/TC nanocomposite (Fig. 6). The highly dispersive of elements distribution verified that there is no impurity in the prepared nanocomposite. It was found that in the item of C (Fig. 6a), Fe (Fig. 6b), N (Fig. 6c), O (Fig. 6d), Zn (Fig. 6e), and Mg (Fig. 6f) which not only exhibited the excellent purity but also disclosed the homogeneous scattering of elements within the ZIF-8/GO/MgFe_2_O_4_/TC. Eventually, Fig. 6g shows the homogeneous arrangement of the elements throughout the structure. Also, SEM imaging in Fig. 6h (10 nm) revealed a few micrometer-sized precipitates, which could not be seen in the SEM images (Fig. 4) due to lower sampling depth.

### Brunauer–Emmett–Teller techniques

**Figure 7:**
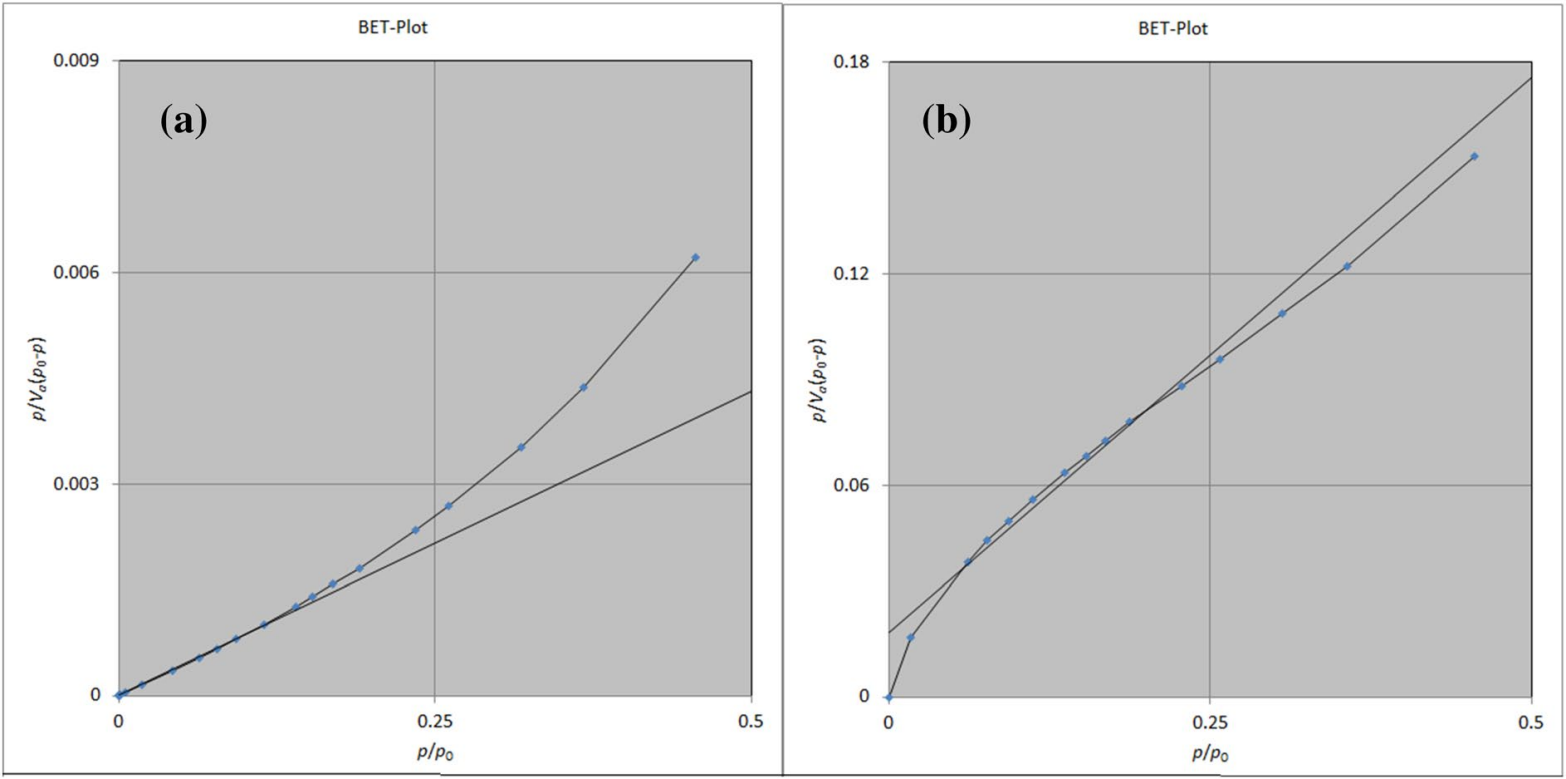
BET-plot of ZIF-8/GO/MgFe_2_O_4_ **(a)**, and ZIF-8/GO/MgFe_2_O_4_/TC **(b)**.

The BET absorption technique is a suitable method to calculate the surface area and porosity of the structures. As shown in the BET plots (Fig. 7), the available surface areas were 504.56 m^2^g^−1^ (Fig. 7a) and 131.06 m^2^ g^−1^ (Fig. 7b) before and after loading TC, respectively. The pore capacity of the cavities in the ZIF-8/GO/MgFe_2_O_4_ is 0.3378 cm^3^ g^−1^ which has been decreased to 0.059053 cm^3^ g^−1^ after encapsulation of the TC. This information confirm that the encapsulation of the TC in the cages of ZIF-8/GO/MgFe_2_O_4_.

**Figure 8:**
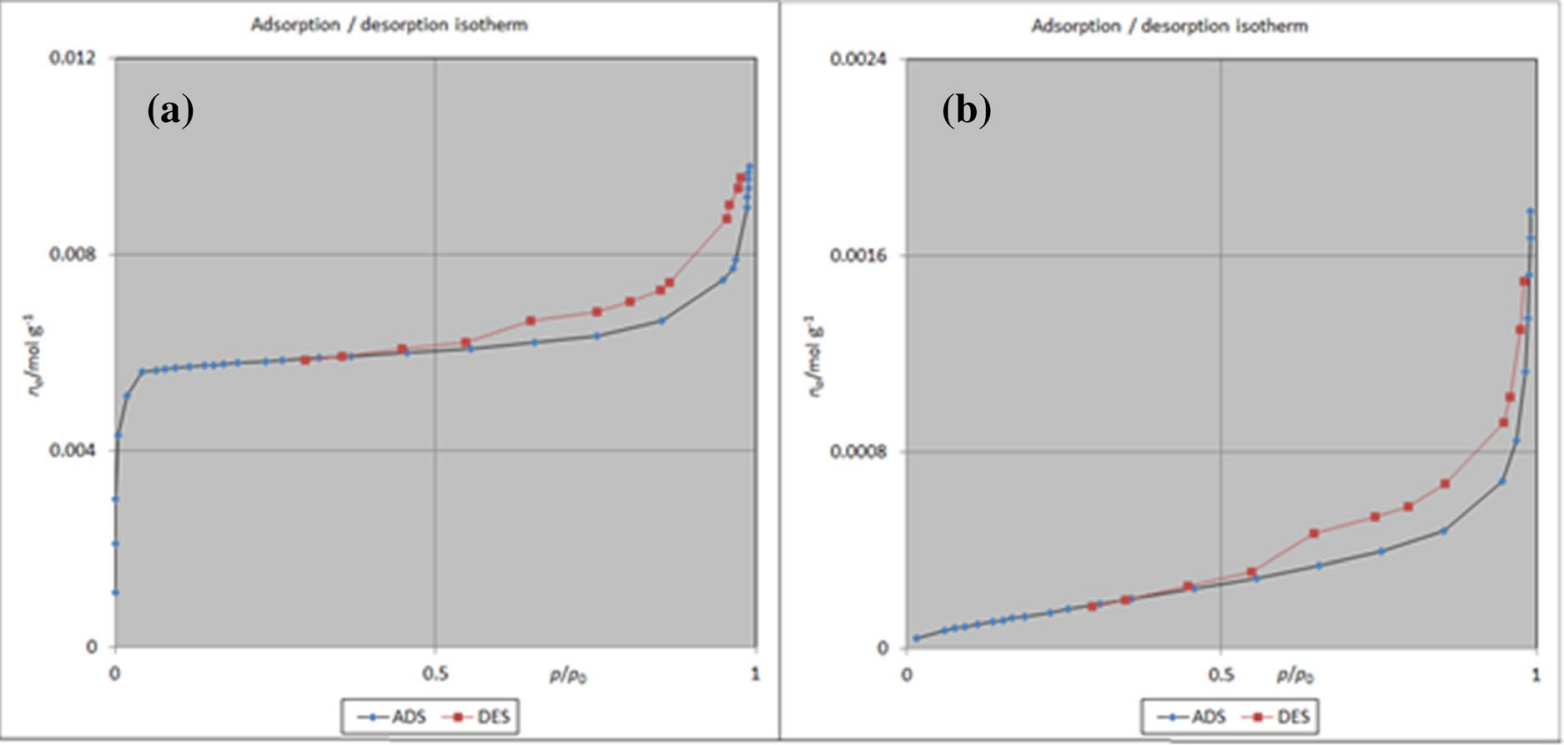
Adsorption / desorption of ZIF-8/ GO/MgFe_2_O_4_ **(a)**, and ZIF-8/GO/MgFe_2_O_4_/TC **(b)**.

**Figure 9:**
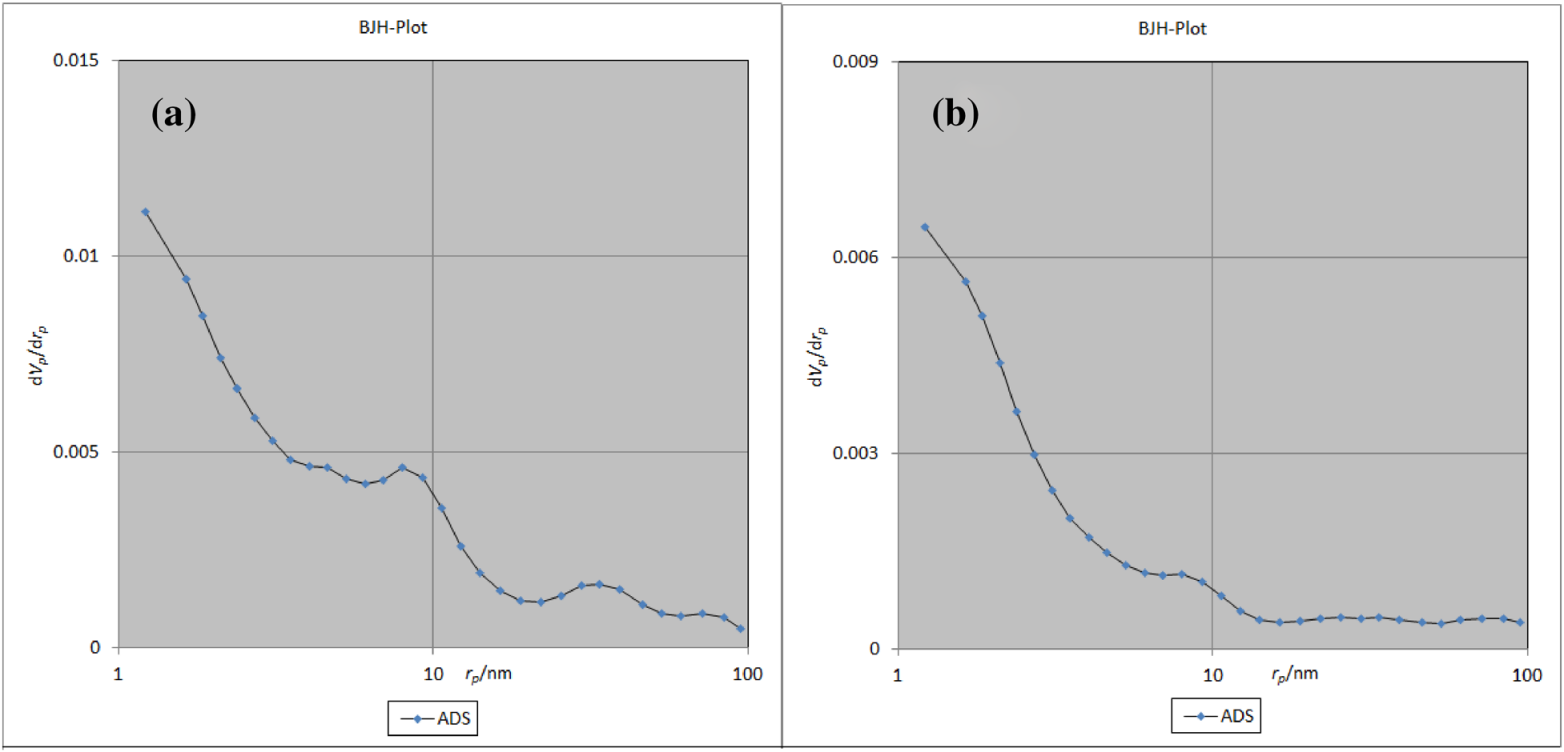
BJH-plot of ZIF-8/GO/MgFe_2_O_4_ **(a)**, and ZIF-8/GO/MgFe_2_O_4_/TC **(b)**.

Also, BET analysis was applied to investigate the adsorption/desorption of ZIF-8/GO/MgFe_2_O_4_ before and after loading with by TC. As displayed in Fig. 8, the adsorption–desorption isotherm of ZIF-8/GO/MgFe_2_O_4_ is type I^28^. The results of the BJH technique indicate that the average pore diameter are 1.21 nm, before and after drug loading (Fig. 9).

### X-ray diffraction analysis

**Figure 10:**
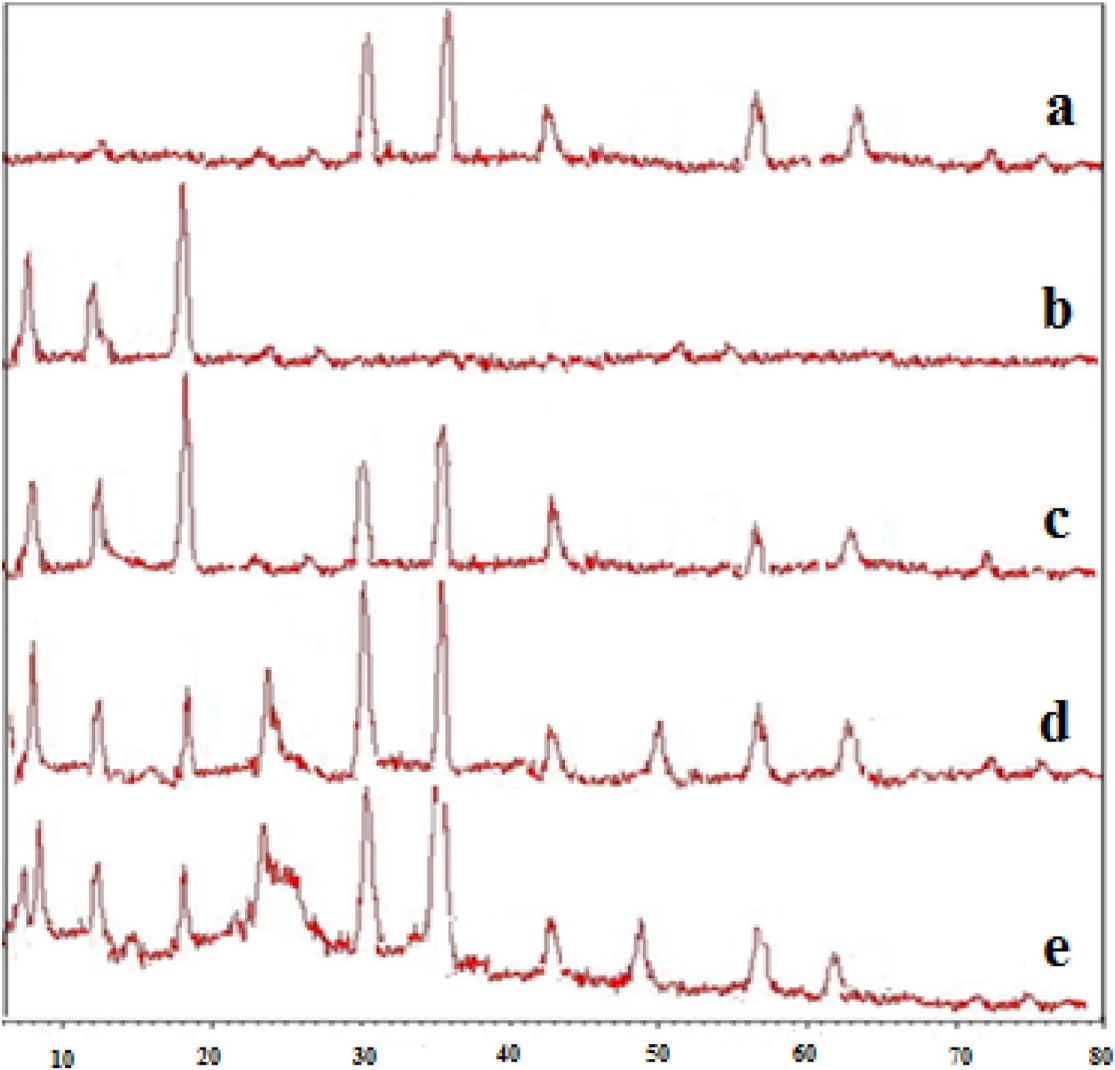
XRD patterns of MgFe_2_O_4_ **(a)**, ZIF-8 **(b)**, ZIF-8/MgFe_2_O_4_ **(c)**, ZIF-8/GO/MgFe_2_O_4_ **(d)**, and ZIF-8/MgFe_2_O_4_/GO/TC **(e)**.

XRD technique was used to verify the structure of the prepared nanoparticles. Figure 10a shows XRD pattern of the MgFe_2_O_4_ nanoparticles which includes diffraction peaks at 30.1°, 35.2°, 43.1°, 57.0°, and 62.6° (2θ°) verifying the successful preparation of crystalline phase in the MgFe_2_O_4_^29^. In XRD pattern of ZIF-8 the peaks at 7.5°, 12.5°, and 18° (2θ°) show the formation ZIF-8 metal–organic frameworks (Fig. 10b). The XRD analysis associated with ZIF-8/MgFe_2_O_4_ showed that both of the synthesized materials were crystalline and their shapes is consistently based on single crystal techniques (Fig. 10c). The study of the XRD pattern of the ZIF-8/GO/MgFe_2_O_4_ (Fig. 10d), revealed that the presented peaks at 9.3° and 11.3° (2θ°) indicated the satisfactory cordination of the GO in the ZIF-8/MgFe_2_O_4_ composite. The XRD pattern of the final structure including ZIF-8/MgFe_2_O_4_/GO/TC (Fig. 10e), conforms the peaks at 30.1°, 35.2°, 43.1°, 57.0°, and 62.6° (2θ°) indicate the formation of the MgFe_2_O_4_ NPs. Also, the presence peaks at 7.5°, 12.5°, and 18° (2θ°) show the formation of the ZIF-8 frameworks, as well as, the peaks at 9.3° and 11.3° (2θ°) indicate the construction of the GO^30^. From this information it can be concluded that after loading by TC, the crystallite structure of ZIF-8/MgFe_2_O_4_/GO is preserved.

### Thermalgravimetric analysis

**Figure 11:**
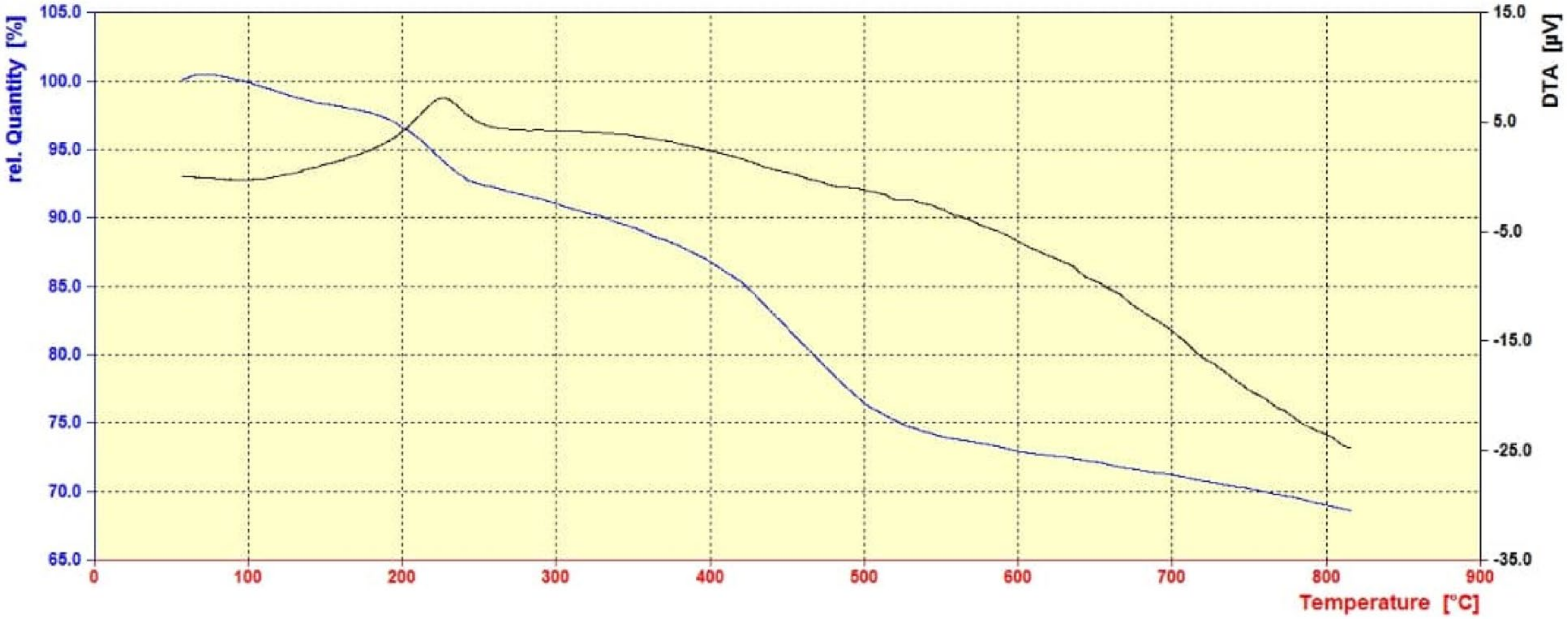
TGA analysis of the ZIF-8/GO/MgFe_2_O_4_/TC.

TGA analysis of ZIF-8/GO/MgFe_2_O_4_/TC shows around 7% weight loss related to the vaporization of solvents in the 0–220 °C (Fig. 11). The weight loss about 8%, beginning from 220 to 400 °C is recognized to the devastation of TC. Moreover, the breakdown of the MOF was detected at the range of 400–500 °C. Eventually, destruction up to 800 °C is attributed to the GO^31^.

### Study on tetracycline release

**Figure 12:**
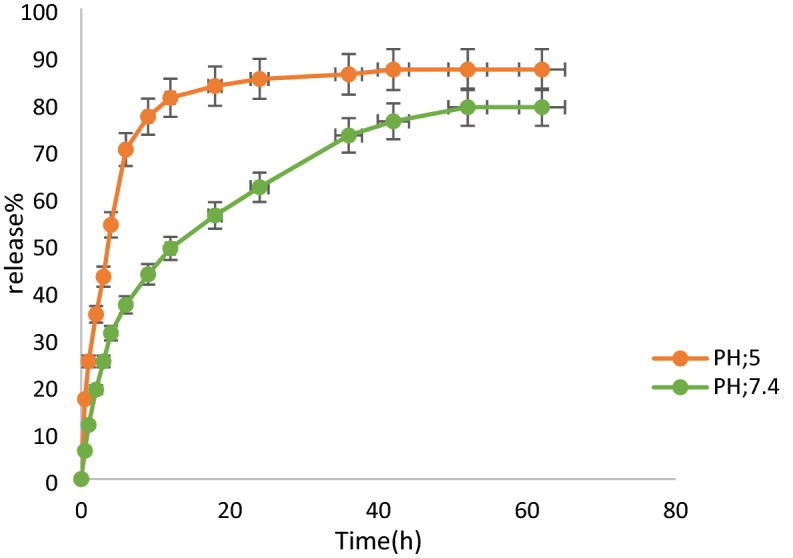
TC release from ZIF-8/GO/MgFe_2_O_4_ in acetate buffer and phosphate buffer saline.

To calculate the amount of TC loading on the nanocarrier, various concentrations of the TC were made in two buffers (pH: 5 and pH: 7.4) solutions and the absorptions were got by a UV–Vis device at a wavelength of 360 nm. Next, their calibration curves were plotted. The release diagrams at pH: 5 and pH: 7.4 are shown in Fig. 12. As displayed, the stability of ZIF-8/GO/MgFe_2_O_4_/TC nanocarrier at pH: 5, which was quickly destroyed in comparison with pH: 7.4, probably due to the destruction of the structure the TC release at pH: 5 was very quicker than pH: 7.4.

The achieved consequences from Fig. 12 display that the percentage of the TC release at pH: 5 increased to 80% in the first 6 h. Although, the release of the TC at physiological of the body (pH: 7.4) was near 76% over 40 h.

### The results of the antimicrobial tests

**Figure 13:**
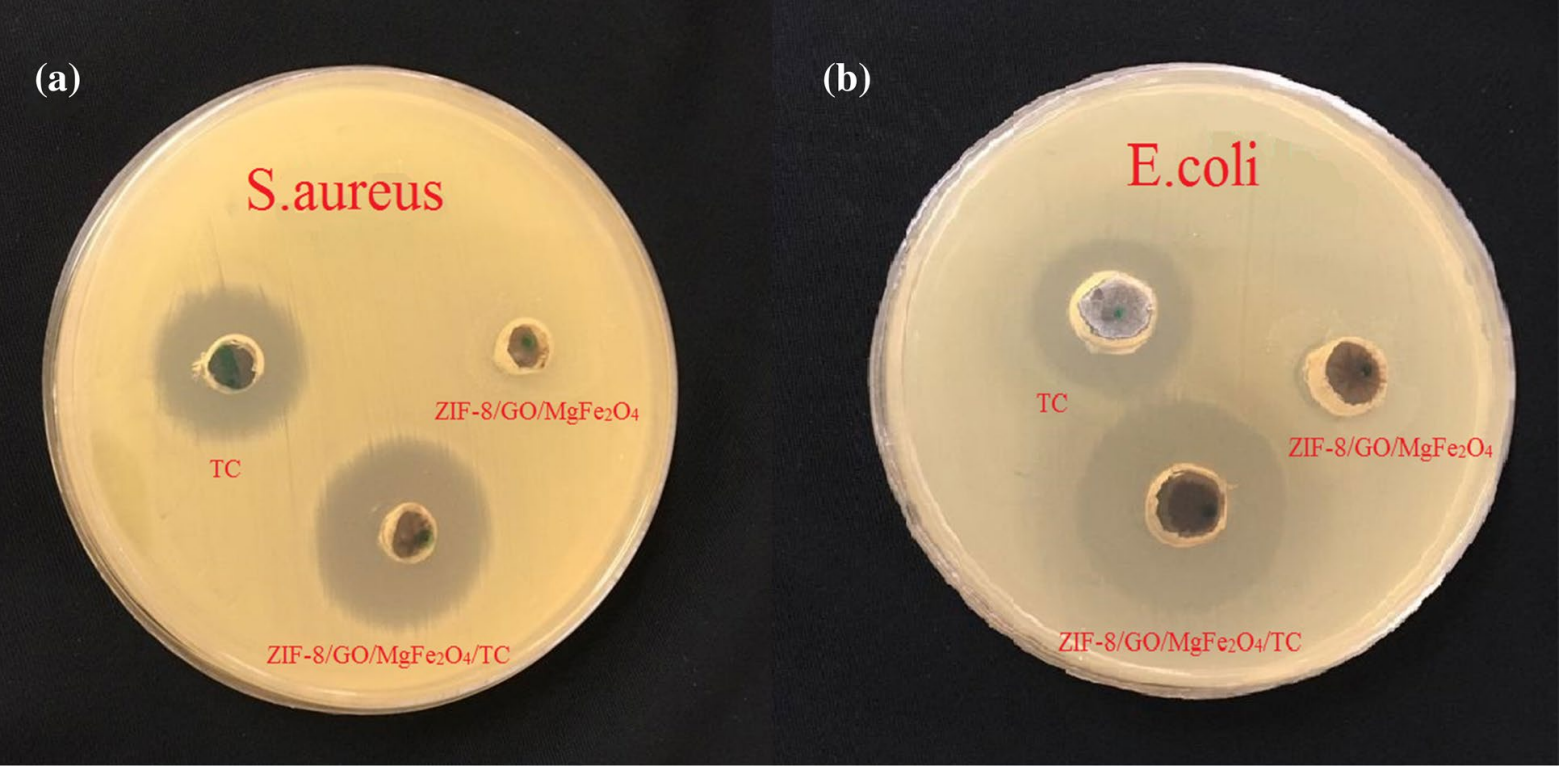
Results of antibacterial tests **(a) **plate containing *Staphylococcus aureus*, and **(b)** plate containing *Escherichia coli* bacteria.

Antimicrobial activities of the tetracycline and the prepared structures were performed on standard strains such as of *S. aureus* and *E. coli*. As indicated in Fig. 13 and Table 1, among various structures such as pure tetracycline, ZIF-8/GO/MgFe_2_O_4_, and ZIF-8/GO/MgFe_2_O_4_/TC, the superlative outcomes were got in the presence of ZIF-8/GO/MgFe_2_O_4_/TC as a strong antibacterial agent. In this research, the diameter of the zone of inhibition was evaluated after incubation to conclude the antimicrobial activity. The results revealed that TC loaded on the MOF was as well as inhibited the development of both bacteria. Therefore, it can be established that the use of ZIF-8/GO/MgFe_2_O_4_ as nanocarrier can increase the antibacterial activities of the TC.

**Table 1:** The antibacterial activity of TC, ZIF-8/GO/MgFe_2_O_4_, ZIF-8/GO/MgFe_2_O_4_/TC.

Bacterium	TC	ZIF-8/GO/MgFe_2_O_4_	ZIF-8/GO/MgFe_2_O_4_/TC
*E.coli*	17 mm	0	22 mm
*S.aureus*	18 mm	0	25 mm

As shown in Table 1, inhibition zone of 22 mm against *E. coli* and 25 mm related to *S. aureus*, such results are obtained when the tetracycline of an inhibitory behavior is shown against *S. aureus* and *E. coli*. With an inhibition zone of 17 mm and 18 mm, respectively.

## Conclusion

The outcomes of this study revealed that the novel nanostructure including ZIF-8/GO/MgFe_2_O_4_ was successfully synthesized based on the characterization and structure elucidation by SEM, EDX/Mapping, XRD, BET, FT-IR, and TGA analysis. In addition, the tetracycline as an antibiotic drug was encapsulated into the ZIF-8/GO/MgFe_2_O_4_ with high loading of 90%, due to the porous nanocomposite, great surface area and cavities in the structure of nanocarrier. In addition, the TC release from the nanocomposite were 88% and 92%, phosphate buffer saline and acetate buffer, respectively. In order to evaluate the antibacterial activities of the prepared composites toward pure tetracycline, the results of by agar well diffusion showed that the antibacterial activity of ZIF-8/GO/MgFe_2_O_4_/TC is more than ZIF-8/GO/MgFe_2_O_4_ and TC.
